# Co morbidities of Myofascial Neck Pain among Information Technology Professionals

**DOI:** 10.1186/s40557-014-0021-4

**Published:** 2014-09-03

**Authors:** Mathankumar Mohandoss, Deepak Sharan, Rameshkumar Ranganathan, Jeena Jose

**Affiliations:** 1 Department of Physiotherapy, RECOUP Neuromusculoskeletal Rehabilitation Centre, # 312, 10th Block, Further Extension of Anjanapura Layout, Bangalore 560062, Karnataka, India; 2 Department of Orthopedic Surgery and Rehabilitation, RECOUP Neuromusculoskeletal Rehabilitation Centre, # 312, 10th Block, Further Extension of Anjanapura Layout, Bangalore 560062, Karnataka, India

**Keywords:** Neck pain, Co-morbidities, Low back pain, Thoracic outlet syndrome, Fibromyalgia, Myofascial pain syndrome

## Abstract

**Objectives:**

The objective of this study was to identify the musculoskeletal co-morbidities of neck pain of myofascial origin among IT professionals.

**Methods:**

A retrospective report analysis of 5357 IT professionals from various IT companies in India was conducted. Demographic details, type and intensity of the musculoskeletal problems, employee feedbacks on status of musculoskeletal health and physician’s diagnosis were analysed. Descriptive statistics were used to describe the age, gender, body area affected and nature of work. Chi square test was used to find the association between musculoskeletal co-morbidities and myofascial neck pain (MNP).

**Results:**

The study participants were predominantly males (71%). 41% of the population used laptops, 35% desktops and 24% both. Neck pain was the commonest reported symptom, followed by low back, shoulder and arm pain respectively. Statistical analysis also revealed that low back pain and shoulder pain, had a significant association with neck pain. Further analysis revealed that there was a significant association between the presence of MNP and thoracic outlet syndrome (p < 0.001) and fibromyalgia syndrome (p < 0.001). Other than the listed co-morbidities, eye strain was also found to be associated with MNP.

**Conclusions:**

Low back pain and shoulder pain was found to be co morbid symptoms noted among IT professionals with MNP. Thoracic outlet syndrome and fibromyalgia were found to be the most commonly associated disorders with MNP among IT professionals.

## Introduction

Musculoskeletal pain is common among IT professionals [1],[2]. Low back pain is the most common musculoskeletal complaint in the general population, with, in the Netherlands, a one year prevalence rate of 44% [3]. The same national study showed a one year prevalence of 31% for neck complaints, 30% for shoulder complaints, 11% for elbow complaints, and 18% for complaints of the wrist [3]. Various risk factors have been proposed for work related musculoskeletal disorders (WRMSD), but its etiology is still unclear. A new approach to find out the etiology of such disorders is the consideration of co-morbidity [4],[5]. Co-morbidity can be defined as the presence of one or more disorders (or diseases) in addition to a primary disease or disorder, or the effect of such additional disorders or diseases. The underlying basis for such studies is that if there is a presence of two or more diseases simultaneously, they may have a common origin. Reporting of one musculoskeletal complaint along with other musculoskeletal co-morbidity is common in literature. Neck pain is one of the most common musculoskeletal disorders (MSD) in the general population, with a 1-year point prevalence of approximately one-third of adults [6]. Co-morbidity studies of MSD have been the third most frequently studied index disease (13%) among all other diseases. A high prevalence of neck pain has been reported among IT professionals. A study carried out by Sharan et al. found that along with neck pain (64.9%), low back pain (56.5%), shoulder pain (42.1%), arm pain (34.5%) and wrist pain (19.8%) were prevalent among the IT professionals in India [2]. It was also reported that myofascial pain syndrome (MPS) was the commonest diagnosis in subjects with neck pain [2]. Given such a high prevalence it is not surprising that a subject suffering from neck pain may often experience other musculoskeletal complaints. Previous studies have found that presence of a MSD other than neck pain, mostly work related or non specific, is a predictor of poor prognosis in neck pain [7]–[9]. Keeping that in mind a study was conducted to identify the musculoskeletal co-morbidities of myofascial neck pain (MNP) among IT professionals in India.

## Materials and Methods

### Study design, setting and population

A retrospective report analysis was conducted in which records of 6563 employees from registers of on-site occupational health clinics situated in different IT companies at three cities (Bangalore, Delhi and Hyderabad) in India were analysed. All the employees engaged in the use of computers (laptop, desktop or both) as a part of their regular work, and visited these on-site clinics for treatment of different MSD. Informed consent was obtained from all the participants before evaluation.

### Study duration and data collection

All the reports from the year 2005 to 2011 were reviewed. Reports included demographic data, chief complaints, diagnosis, treatment given and subjective feedbacks. Demographic data in the reports included age, gender, duration of computer usage per day, and the type of use (Laptop/Desktop/Both). Data regarding type and severity of the musculoskeletal problems were collected from the reports of an orthopaedic and rehabilitation physician who visited all the on-site clinics. A single orthopaedic and rehabilitation physician performed the evaluation and diagnosis of WRMSD’s was made based on time rule. MPS was diagnosed based on Simon’s criteria [10]. Employee feedbacks were also used for evaluating the status of their musculoskeletal health.

### Statistical analysis

Descriptive statistics like mean, percentage and frequency were used to describe the age, gender, body area affected and nature of work. Chi square test was used to find the association between musculoskeletal co-morbidities and neck pain. The data were analysed using statistical software SPSS version 17.0.

## Results

### Demography

Out of the 6563 reports only 5357 were included as the rest of the data was incomplete. 71.5% of the subjects were male and 28.5% female. The mean age of the male and female subjects was 31.10 ± 5.99 years and 29.68 ± 5.59 years respectively. 41% of the population used laptops, 35% desktops and 24% both. The statistical analysis revealed that the distribution of the use of laptop and desktop was almost similar (Odds Ratio: 1.21, 95% Confidence Interval: 0.70 to 2.1). 81.6% of the population was working for 8 to 12 hours in a day. Demographic characteristics of the population were described in Table 1.

**Table 1:** Demographic characteristics of the population (n = 5357)

**Variables**	**n**	**%**
**Gender**		
Male	3831	71.5
Female	1526	28.5
**Age group**		
<25 Years	569	10.6
25 to 35	3822	71.3
36 to 45	840	15.7
46 to 55	117	2.2
>56 years	9	0.2
**Hours of computer work/day**		
< 8 hours	489	9
8 to 12 hours	4371	81.6
>12 hours	497	9.4
**Job category**		
Managerial	1500	28
Software Engineer	1446	27
Application Engineer	1179	22
Analyst	804	15
Human Resource	161	3
Others	267	5
**Type of computer used**		
Desktop	1872	35
Laptop	2187	41
Both	1298	24

### Distribution of symptoms

**Figure 1:**
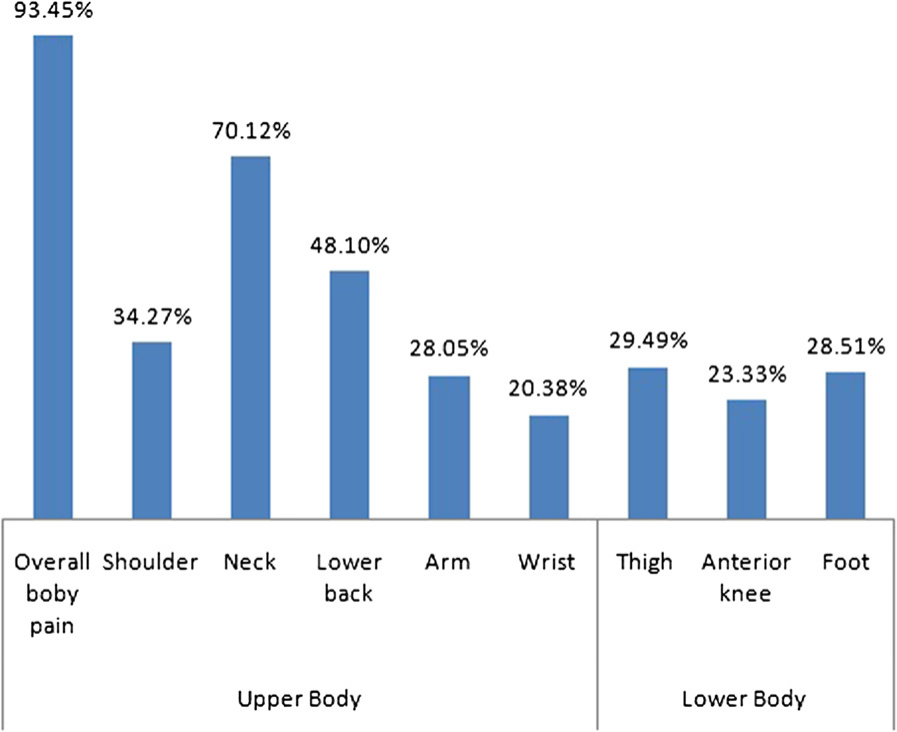
Regional distribution of pain among females.

**Figure 2:**
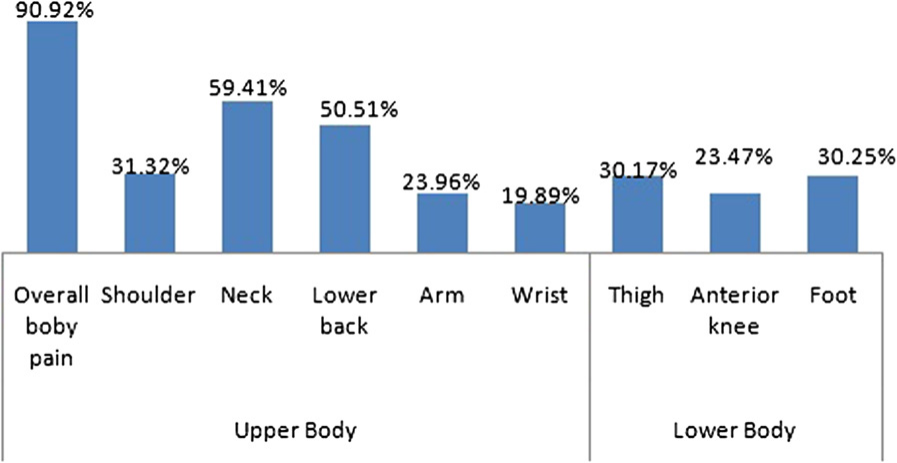
Regional distribution of pain among males.

The distribution of musculoskeletal pain was presented in Figures 1 and 2. Among the body region affected, musculoskeletal discomfort of neck, lower back, shoulder and arm had higher prevalence as compared with other body parts. Statistical analysis also revealed that neck pain among females was significantly (Odds Ratio: 2.4; 95% Confidence Interval: 1.34 to 4.29) higher as compared to males.

### Diagnosis of MSD

**Figure 3:**
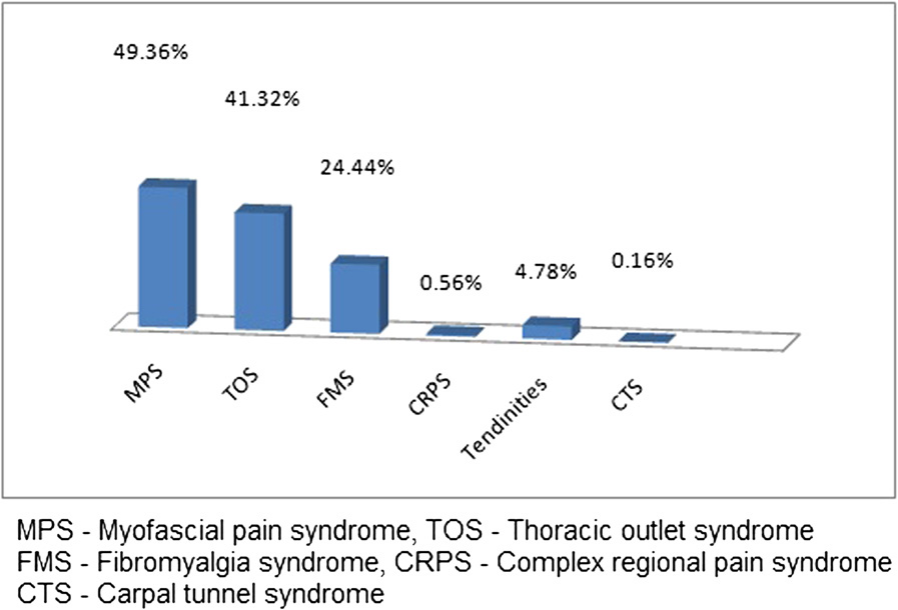
MSD based on diagnosis among females.

**Figure 4:**
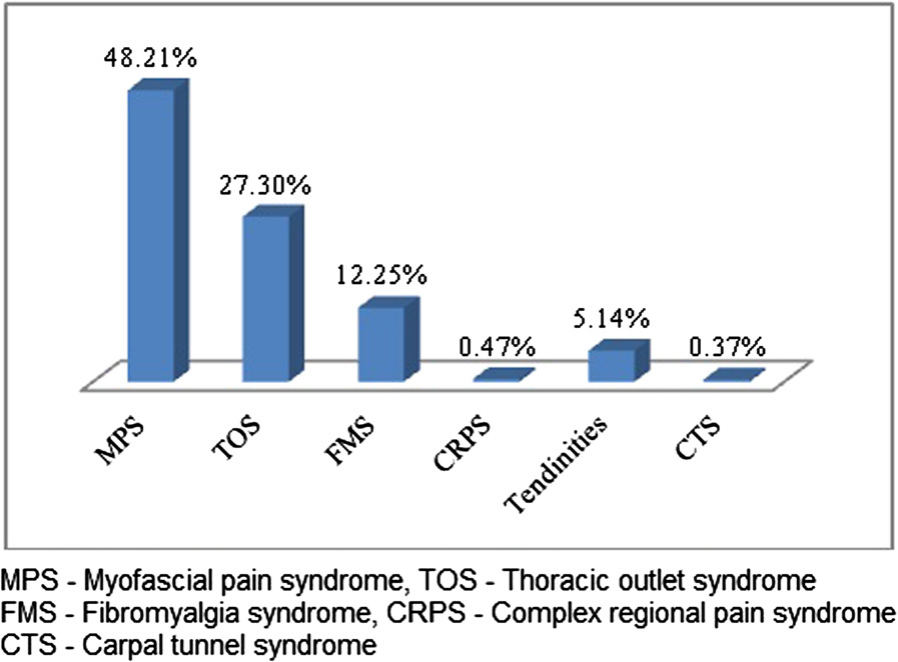
MSD based on diagnosis among males.

Myofascial Pain Syndrome was found to be the commonest MSD that led to pain among both the genders. Thoracic Outlet Syndrome (TOS) and Fibromyalgia Syndrome (FMS) were the other common MSD’s noted among the study population. Distribution of MSD based on diagnosis was presented in Figures 3 and 4.

### Co-morbidities

Chi-square analysis revealed that neck pain was significantly associated with low back pain (p < 0.001), shoulder pain (p < 0.001) and forearm and wrist pain (p < 0.001). Detailed results of various variables and its association with co-morbid symptoms were presented in Table 2. Apart from all the upper extremity musculoskeletal discomfort, eye strain was also found to be comorbid with neck pain (Males – 25.18% and Females – 27.66%).

**Table 2:** Association of various variables with co-morbid symptom and disorder among subjects with myofascial neck pain (n = 3346)

**Variable**	**Co-morbid symptom**						**Co-morbid disorder**			
	**Low back pain**		**Shoulder pain**		**Forearm & wrist pain**		**TOS**^**ф**^		**FMS**^**Ὼ**^	
	**n (%)**	p-value*	**n (%)**	p-value*	**n (%)**	p-value*	**n (%)**	p-value*	**n (%)**	p-value*
**Gender**										
Male (n = 2276)	1064 (47)	0.75	921 (41)	0.47	553 (24)	0.34	913 (40)	0.005^#^	378 (17)	1.7E-05^#^
Female (n = 1070)	494 (46)		447 (42)		244 (23)		484 (45)		244 (23)	
**Age group**										
<25 years (n = 362)	184 (51)	0.004^#^	156 (43)	0.6	65 (17)	0.005^#^	104 (28)	0.00^#^	50 (13)	0.014
25 to 35 (n = 2398)	1126 (47)		975 (41)		564 (24)		1013 (42)		440 (18)	
36 to 45 (n = 526)	218 (41)		204 (38)		145 (27)		236 (44)		119 (22)	
46 to 55 (n = 55)	16 (29)		24 (44)		16 (29)		28 (51)		7 (13)	
>56 years (n = 5)	2 (40)		1(20)		3 (60)		3 (60)		1 (20)	
**Computer work/day**										
< 8 hours (n = 310)	61(20)	0.00^#^	134 (43)	0.00^#^	29 (9.4)	0.00^#^	52 (17)	0.00^#^	18 (6)	0.00^#^
8 to 12 hours (n = 2726)	1262 (46)		1105 (41)		658 (24)		1206 (43)		533 (19)	
>12 hours (n = 310)	204 (66)		186 (60)		73 (25)		90 (29)		31(10)	
**Computer used**										
Desktop (n = 1170)	677(58)	0.00^#^	625 (53)	0.00^#^	255 (22)	0.1	353(30)	0.00^#^	107 (10)	0.00^#^
Laptop (n = 1396)	629 (45)		589 (42)		342 (25)		596 (43)		282 (20)	
Both (n = 780)	252 (32)		154 (20)		200 (26)		448 (57)		233 (30)	

ф- Thoracic outlet syndrome.

Ὼ- Fibromyalgia syndrome.

*- Calculated using the Chi square test.

^#^- Variables with significant association.

TOS was found to be comorbid with MNP in both males (40.10%) and females (45.23%). On the other hand co-morbidity of FMS with MNP was found to be 16.61% for males and 22.80% for females respectively. Further analysis revealed that there was a significant association between MNP and TOS (p < 0.001) and FMS (p < 0.001). Various variables and its correlation with TOS and FMS are presented in Table 2.

## Discussion

Musculoskeletal co-morbidity is common in the field of occupational health. The addition of co-morbidities to various WRMSD leads to an increment in the absenteeism [11]–[13]. The co-morbidity of neck and low back pain, reported as 68%, affected health care utilisation and absenteeism [13],[14]. In another study confounding co-morbidity of low back pain and neck pain was reported and low back pain was considered as predictor of neck pain [15],[16]. The present study also revealed a similar result.

The present study revealed that low back pain, shoulder pain, forearm and wrist pain were co morbid with MNP among the IT professionals in India. There was a strong correlation between these symptoms which was suggestive of awkward posture as a risk factor for WRMSD. Eye strain was found to be the other common co morbid symptom noted among IT professionals with MNP. The study suggested the presence of symptoms in multiple regions with longer working hours, in both genders irrespective of their age and type of computer usage. Low back pain as a co-morbid symptom of neck pain was more common in younger age groups and in subjects who worked for longer hours. Forearm and wrist pain as a co-morbid symptom of neck pain was more common in older age groups and in subjects who worked for longer hours. This is suggestive of static loading as a possible risk factor for low back pain and abnormal posture as a possible risk factor for wrist pain.

Co-morbidity of neck pain and FMS is well reported in the literature [17],[18]. The present study also corroborated these findings. It has been reported that psychological symptoms were higher among the co-morbid patients [16]. A study on the mental-physical co-morbidity and its relation with disability suggested that with increasing number of co-morbidities the psychosocial risk increased which ultimately led to reduced productivity and poorer health [19].

Among workers with low back pain, subjects with high pain intensity or disabling low back pain are more likely to have musculoskeletal co-morbidity [12]. The present study involving IT professionals showed a high prevalence of other musculoskeletal co-morbidities with neck pain. For occupational health practitioners the finding of a high prevalence of co-morbidities is important to consider when implementing workplace interventions aimed at the reduction of specific musculoskeletal complaints, since the controls for one musculoskeletal complaint may impact adversely on another musculoskeletal complaint [12].

Myofascial pain syndrome was found to be the commonest cause for neck pain. TOS and FMS were the commonest disorders associated with MNP among the same population. TOS as a co morbidity of MNP was more common in older age groups and in subjects who worked for longer hours, which is suggestive of MNP being a predictor of TOS. It is possible that early detection and treatment of MNP could prevent occurrence of TOS. Further research is recommended to identify the root cause of such co-morbidities.

### Consent

Written informed consent was obtained from the all the subjects before the evaluation for publication of this report.

## Competing interests

The authors declare that they have no competing interests.

## Authors’ contributions

MM participated in the conception and design, statistical analysis, drafting and critical review of the manuscript. DS Participated in conception and design, assessment of subjects, critical review of manuscript. RR participated in data collection and critical review of the manuscript. JJ participated in data collection and critical review of the manuscript. All authors read and approved the final manuscript.

## References

[B1] Sharan D Parijat P Sasidharan AP Ranganathan R Mohandoss M Jose J Workstyle risk factors for work related musculoskeletal symptoms among computer professionals in India J Occup Rehabil 2011 21 4 520 525 10.1007/s10926-011-9294-4 21328059

[B2] Sharan D Ajeesh PS Rameshkumar R Jose J Risk factors, clinical features and outcome of treatment of work related musculoskeletal disorders in on-site clinics among IT companies in India Work 2012 41 Suppl 1 5702 5704 22317658 10.3233/WOR-2012-0924-5702

[B3] Picavet HS Schouten JS Musculoskeletal pain in the Netherlands: prevalences, consequences and risk groups, the DMC (3)-study Pain 2003 102 167 178 10.1016/s0304-3959(02)00372-x 12620608

[B4] Gijsen R Hoeymans N Schellevis FG Ruwaard D Satariano WA Van-den BGAM Causes and consequences of co-morbidity: a review J Clin Epidemiol 2001 54 7 661 674 10.1016/S0895-4356(00)00363-2 11438406

[B5] TRS 919 2003 World Health Organization, Geneva, Switzerland

[B6] Croft PR Lewis M Papageorgiou AC Thomas E Jayson MI Macfarlane GJ Silman AJ Risk factors for neck pain: a longitudinal study in the general population Pain 2001 93 3 317 325 10.1016/S0304-3959(01)00334-7 11514090

[B7] Carroll LJ Hogg-Johnson S Côté P van der Velde G Holm LW Carragee EJ Hurwitz EL Peloso PM Cassidy JD Guzman J Nordin M Haldeman S Course and prognostic factors for neck pain in workers: results of the Bone and Joint Decade 2000–2010 Task Force on Neck Pain and Its Associated Disorders Spine 2008 33 Suppl 4 S93 S100 10.1097/BRS.0b013e31816445d4 18204406

[B8] McLean SM, May S, Moffett J, Sharp DM, Gardiner E: **Prognostic factors for progressive non-specific neck pain: a systematic review.** *Phys Ther Rev* 2007, **12**(3):220.

[B9] Walton DM Carroll LJ Kasch H Sterling M Verhagen AP Macdermid JC Gross A Santaguida PL Carlesso L An overview of systematic reviews on prognostic factors in neck pain: results from the international collabration on neck pain Open Orthop J 2013 7 494 505 10.2174/1874325001307010494 24115971 PMC3793581

[B10] Simons DG Diagnostic criteria of myofascial pain due to trigger points J Musculoskelet Pain 1999 7 1/2 111 120 10.1300/J094v07n01_11

[B11] Alexopoulos EC, Stathi IC, Charizani F: **Prevalence of musculoskeletal disorders in dentists.** *BMC Musculoskelet Disord* 2004, **5:**16. 10.1186/1471-2474-5-16 PMC441388 15189564

[B12] Alexopoulos EC, Tanagra D, Konstantinou E, Burdorf A: **Musculoskeletal disorders in shipyard industry: prevalence, healthcare use, and absenteeism.** *BMC Musculoskelet Disord* 2006, **7:**88. 10.1186/1471-2474-7-88 PMC1676002 17125504

[B13] IJzelenberg W Burdorf A Impact of musculoskeletal co-morbidity of neck and upper extremities on healthcare utilization and sickness absence for low back pain Occup Environ Med 2004 61 806 810 10.1136/oem.2003.011635 15377765 PMC1740669

[B14] Haukka E Arjas PL Solovieva S Ranta R Viikari-Juntura E Riihimäki H Co-occurrence of musculoskeletal pain among female kitchen workers Int Arch Occup Environ Health 2006 80 2 141 148 10.1007/s00420-006-0113-8 16688464

[B15] Hill J Lewis M Papageorgiou AC Predicting persistent neck pain: A 1-year follow-up of a population cohort Spine 2004 29 1648 1654 10.1097/01.BRS.0000132307.06321.3C 15284511

[B16] Hoving JL De Vet HCW Twisk JWR Prognostic factors for neck pain in general practice Pain 2004 110 639 645 10.1016/j.pain.2004.05.002 15288404

[B17] Jordan SE Ahn SS Gelabert HA Differentiation of thoracic outlet syndrome from treatment-resistant cervical brachial pain syndromes: development and utilization of a questionnaire, clinical examination and ultrasound evaluation Pain Physician 2007 10 3 441 452 17525778

[B18] Cakit BD Taskin S Nacir B Unlu I Genc H Erdem HR Co-morbidity of fibromyalgia and cervical myofascial pain syndrome Clin Rheumatol 2010 29 4 405 411 10.1007/s10067-009-1342-5 20066449

[B19] Scott KM Korff MV Alonso J Angermeyer MC Bromet E Fayyad J de Girolamo G Demyttenaere K Gasquet I Gureje O Haro JM He Y Kessler RC Levinson D Mora MEM Browne MO Ormel J Villa JP Watanabe M Williams D Mental-Physical Co-morbidity and its relationship with disability: results from the world mental health surveys Psychol Med 2009 39 1 33 43 10.1017/S0033291708003188 18366819 PMC2637813

